# Reflex somatic testing for the detection of *FGFR* alterations in urinary tract carcinomas: A dual-institutional experience

**DOI:** 10.1093/ajcp/aqaf108

**Published:** 2025-10-16

**Authors:** Ngoc-Nhu Jennifer Nguyen, Ekaterina Olkhov-Mitsel, Kenneth J Craddock, Trevor A Flood, Michelle R Downes

**Affiliations:** Department of Laboratory Medicine and Pathobiology, University of Toronto, Toronto, Ontario, Canada; Department of Anatomic Pathology, Precision Diagnostics & Therapeutics Program, Sunnybrook Health Sciences Centre, Toronto, Ontario, Canada; Department of Anatomic Pathology, Precision Diagnostics & Therapeutics Program, Sunnybrook Health Sciences Centre, Toronto, Ontario, Canada; Department of Laboratory Medicine and Pathobiology, University of Toronto, Toronto, Ontario, Canada; Department of Anatomic Pathology, Precision Diagnostics & Therapeutics Program, Sunnybrook Health Sciences Centre, Toronto, Ontario, Canada; Department of Pathology and Laboratory Medicine, The Ottawa Hospital, Ottawa, Ontario, Canada; Department of Laboratory Medicine and Pathobiology, University of Toronto, Toronto, Ontario, Canada; Department of Anatomic Pathology, Precision Diagnostics & Therapeutics Program, Sunnybrook Health Sciences Centre, Toronto, Ontario, Canada

**Keywords:** urinary tract carcinoma, *FGFR*, next-generation sequencing

## Abstract

**Objective:**

To describe the prevalence and clinicopathologic associations of *FGFR*-altered urinary tract carcinomas in institutions incorporating reflex testing.

**Methods:**

Next-generation sequencing was prospectively performed on urinary tract carcinomas for the detection of *FGFR1-4* alterations at 2 tertiary care centers (2021-2025), using the Oncomine Comprehensive Assay (OCA) v3 DNA and OCA Plus RNA. Reflex testing was conducted on metastatic and/or advanced (pT3/4) carcinomas.

**Results:**

The cohort included 366 patients (239 lower tract carcinomas, 72 upper tract carcinomas, and 55 metastases). Median age was 72.5 years (range, 36-97). Fifty-nine (16.1%) tumors were *FGFR*-altered. Forty-nine (13.4%) patients with actionable *FGFR* alterations (33 *FGFR3* mutations, 13 *FGFR3* fusions, and 3 *FGFR2* mutations) were all 55 years or older (*P* = .097). The prevalence of actionable *FGFR* alterations was significantly higher in upper vs lower tract carcinomas (23.8% vs 13.8%, *P* = .007) and in lung metastases vs other metastatic sites (57.1% vs 10.4%, *P* = .002). A higher frequency was also seen in metastases vs primary tumors (16.4% vs 12.9%), although this difference did not reach statistical significance (*P* = .482). Actionable *FGFR* alterations were observed in urothelial carcinoma not otherwise specified (40/261) and in urothelial carcinoma with squamous differentiation (6/43), micropapillary features (2/11), or nested features (2/7).

**Conclusions:**

The detection rate for *FGFR1-4* alterations in a real-world, dual-institution cohort of urinary tract carcinomas was reported.

**KEY POINTS**

- Based on a real-world, dual-institution cohort of urinary tract carcinomas, this study is the first to report the prevalence of *FGFR1-4* alterations and the first to assess the benefit of integrating reflex somatic testing in the clinical workflow.
- *FGFR1-4* alterations were present in 16.1% of patients, and actionable alterations were seen in 13.4% of patients. Upper tract urothelial carcinomas and lung metastases were enriched in *FGFR* alterations.
- Findings showcased in this study may help establish guidelines for predictive somatic *FGFR* testing in urinary tract carcinomas and shape therapeutic strategies.

## INTRODUCTION

It is estimated that in 2024, urinary bladder cancer was responsible for 83 190 new cancer diagnoses and 16 840 cancer-specific deaths in the United States.^1^ The 5-year relative survival rate for urinary bladder cancer is 78% overall but drops to 9% in the metastatic setting.^2^ Distant metastasis is present at diagnosis in 5% of patients with bladder cancer, and the risk of developing metastatic disease is very high in patients with locally advanced bladder cancer.^3^ Platinum-based chemotherapy, immune checkpoint inhibitors, and enfortumab vedotin (an antibody drug conjugate targeting Nectin-4) are used as first- and second-line therapies in advanced or metastatic bladder cancer.^4,5^ For patients who are ineligible or nonresponsive to these therapies, therapeutic options include trastuzumab deruxtecan, a HER2-directed monoclonal antibody, for tumors with HER2 overexpression by immunohistochemistry (3+), and erdafitinib, a pan-fibroblast growth factor receptor (FGFR) inhibitor, for tumors with actionable *FGFR* alterations.^4,5^

FGFR is a tyrosine kinase receptor that can be coded by 1 of 4 genes (*FGFR1-4*).^6-8^ It is activated by the extracellular binding of 1 of 18 fibroblast growth factors, which leads to the phosphorylation of intracellular proteins, including phospholipase C (PLC) γ and FGFR substrate 2 (FRS2).^6-8^ The PLC γ phosphorylation activates protein kinase C, while FRS2 phosphorylation promotes the activation of the Ras/MAPK and PI3K/Akt signaling pathways.^6-8^ These pathways play an essential role in cell proliferation, differentiation, and survival. Activating *FGFR* alterations, common in bladder cancer, can thereby contribute to carcinogenesis.

Loriot et al^9,10^ led phase II and phase III trials (BLC2001) assessing erdafitinib in patients with metastatic or unresectable, locally advanced urothelial carcinoma that progressed after chemotherapy.^11^ Patients included in the studies had select *FGFR3* mutation or *FGFR2/3* fusion. A phase II study showed an overall response rate of 40% among 99 patients, with a median progression-free survival time of 5.5 months and a median overall survival time of 13.8 months.^9^ Complete response was seen in 3% of patients and a partial response in 37% of patients. Prior immunotherapy was associated with a higher response rate to erdafitinib (59%). The phase III trial included 266 patients who experienced disease progression after treatment with immune checkpoint inhibitors.^10,11^ Patients were randomized into the erdafitinib group (n = 136) or the chemotherapy group (n = 130). The median progression-free survival time and the median overall survival time were significantly higher in the erdafitinib group than in the chemotherapy group. While both groups had a similar incidence of grade 3 or 4 adverse events, chemotherapy was associated with more deaths due to these adverse events. This trial demonstrated the overall superiority of erdafitinib compared to chemotherapy in metastatic or advanced urothelial carcinoma.

In 2019, based on the phase II trial led by Loriot and colleagues^^9^^, the US Food and Drug Administration (FDA), followed by Canada’s Drug Agency, approved the use of erdafitinib in patients with metastatic or locally advanced urothelial carcinoma, select hotspot *FGFR3* mutations or *FGFR2/3* fusions, and disease progression within 12 months following platinum-based chemotherapy.^12,13^ In light of this, provincial health authorities in Canada implemented and funded pathologist-driven reflex *FGFR2/3* somatic testing in metastatic/advanced bladder cancer.^14^ Results from the phase III BLC2001 trial^^10^^ prompted the FDA and Canada’s Drug Agency to modify the eligibility criteria in 2024 and 2025, respectively, restricting the administration of erdafitinib to patients with susceptible *FGFR2/3* alterations who are ineligible or did not respond well to immune checkpoint inhibitors.^15,16^

The aim of the current work was to address the paucity of prospective studies evaluating the prevalence and clinicopathologic characteristics of *FGFR*-altered urinary tract carcinomas. A dual-institution experience with somatic *FGFR* testing of urinary tract carcinomas, incorporating both oncologist-driven and pathologist-driven reflex testing, is herein reported.

## METHODS

### Cohort selection

Urinary tract carcinomas were prospectively sequenced for the detection of *FGFR* alterations at 2 tertiary care centers serving a multiethnic population (2021-2025). In addition to somatic testing performed upon oncologists’ requests, pathologist-driven reflex testing was performed on all metastatic and/or locally advanced carcinomas of the urinary tract. Locally advanced carcinomas were defined as pathological tumor stage pT3 or pT4. One formalin-fixed, paraffin-embedded (FFPE) specimen was submitted per patient for next-generation sequencing. The dual-institution cohort comprised both in-house and external specimens from referring institutions.

### Clinicopathologic review

Patient age, sex, histologic type, tumor site, pathological tumor (pT) stage, and pathological nodal (pN) stage were collected. Biomarker reports were reviewed for actionable (tier 1/2) and uncertain significance (tier III) *FGFR* alterations. In addition, for specimens with a readily available variant call format file, an analysis was performed to identify oncogenic (tier 1 or 2) mutations in *PIK3CA*, *TP53*, and *RB1*, which are not clinically reported in either participating hospital. This study was approved by the research ethics board of both institutions.

### Molecular analysis

Manual macrodissection was performed on unstained slides obtained from the FFPE block, as guided by pathologist annotations on the pre- and/or post–hematoxylin and eosin (H&E) slides. Next-generation sequencing was performed on the Ion GeneStudio S5 Prime System, using the Oncomine Comprehensive Assay (OCA) v3 DNA panel and the OCA Plus RNA panel. Quality control metrics for DNA samples included the number of mapped reads (≥3 000 000), the mean depth (≥800), the uniformity (≥80%), and the mean read length (≥75 bp). Quality metrics for RNA samples included the mean read length (≥50 bp), the total number of mapped reads (≥500 000), and the number of reads for each primer pool (≥100,000). *FGFR1-4*, *PIK3CA*, *TP53*, and *RB1* variants were identified using the Ion Reporter v5.18 and interpreted for actionability in accordance with guidelines from the Association for Molecular Pathology (AMP), the American Society of Clinical Oncology (ASCO), and the College of American Pathologists (CAP).^17^ A variant allele frequency (VAF) of 5% and a minimum depth of 500× coverage were required to report DNA variants, while the amplicon read threshold for fusion calling was 200×. One important exception to the coverage requirement was the common hotspot p.S249C mutation, which falls within a chronically low-coverage amplicon, likely due to a GC-rich sequence in the region. For this variant, coverage tended to be between 200 and 500×, while the VAF threshold of 5% still applied for reported variants. Variant annotations were based on the hg19 (GRCh37) genome build, using the MANE Select transcripts (NM_000142.5 for *FGFR* variants).

### Statistical analysis

Descriptive statistics were used to summarize the clinicopathologic characteristics of the cohort and the prevalence of *FGFR* alterations. Comparisons between categorical variables, including sex, histologic type, tumor site, pT stage, and pN stage, were performed using the χ^2^ test or Fisher exact test, as appropriate. The Mann-Whitney *U* test was used for continuous variables. For prevalence calculations, tumors harboring multiple *FGFR* alterations were counted once as positive for an *FGFR* alteration. Urothelial carcinomas exhibiting multiple histologic variants were counted separately for each variant when evaluating histologic correlates. The χ^2^ test was used to compare the frequency of comutations between *FGFR*-altered and *FGFR*-negative specimens. A 2-sided *P* value less than .05 was considered statistically significant. All statistical analyses were conducted using SPSS version 26 (IBM).

## RESULTS

### Cohort characteristics

The dual-institution cohort comprised 366 patients, with a male-to-female ratio of 3.1:1. Median age was 72.5 years (range, 36-97 years). The specimen types were as follows: transurethral resection (n = 126), cystoprostatectomy (n = 85), nephroureterectomy (n = 46), cystectomy (n = 24), kidney biopsy (n = 16), ureterectomy (n = 6), ureter biopsy (n = 4), urethrectomy (n = 2), urethra biopsy (n = 2), and metastasis biopsy (n = 55). Tables 1, 2 and 3 present the clinicopathologic features of the cohort along with the prevalence of *FGFR* alterations. All sequenced tumors were of high grade.

**Table 1:** Association Between Clinicopathological Variables and the Prevalence of *FGFR* Alterations in the Full Study Cohort (N = 366)

Characteristic	N	Actionable (tier 1 or 2) *FGFR* alteration(s), No. (%)	χ^2^ *P* value	Any (tier 1, 2, or 3) *FGFR* alteration(s), No. (%)	χ^2^ *P* value
Age, y			.097		.240
<55	17	0 (0)		1 (5.9)	
≥55	34	49 (14.0)		58 (16.6)	
Sex			.986		.871
Male	276	37 (13.4)		44 (15.9)	
Female	90	12 (13.3)		15 (16.7)	
Primary specimen vs metastatic specimen			.482		.652
Primary specimen	311	40 (12.9)		49 (15.8)	
Metastatic specimen	55	9 (16.4)		10 (18.2)	
Histologic type			.531		.728
Urothelial carcinoma	358	49 (13.7)		58 (16.2)	
Squamous cell carcinoma	5	0 (0)		1 (20.0)	
Adenocarcinoma	3	0 (0)		0 (0)	
Urothelial carcinoma subtype/divergent differentiation			.524		.608
Pure urothelial carcinoma not otherwise specified	261	40 (15.3)		46 (17.6)	
Squamous differentiation	43	6 (14.0)		8 (18.6)	
Glandular differentiation	11	0 (0)		1 (9.1)	
Trophoblastic differentiation	1	0 (0)		0 (0)	
Plasmacytoid features	16	0 (0)		0 (0)	
Micropapillary features	11	2 (18.2)		2 (18.2)	
Nested features	7	2 (28.6)		2 (28.6)	
Neuroendocrine features	7	0 (0)		0 (0)	
Sarcomatoid features	6	0 (0)		0 (0)	
Clear cell features	2	0 (0)		0 (0)	
Microcystic features	1	0 (0)		0 (0)	

**Table 3:** Prevalence of *FGFR* Alterations by Site of Metastasis (n = 55)

Characteristic	N	Actionable (tier 1 or 2) *FGFR* alteration(s), No. (%)	χ^2^ *P* value	Any (tier 1, 2, or 3) *FGFR* alteration(s), No. (%)	χ^2^ *P* value
Site of metastasis			.063		.110
Node	16	2 (12.5)		2 (12.5)	
Liver	14	2 (14.3)		2 (14.3)	
Lung	7	4 (57.1)		4 (57.1)	
Neuro	4	0 (0)		1 (25.0)	
Bone	2	0 (0)		0 (0)	
Other	12	1 (8.3)		1 (8.3)	
Lung vs other sites of metastasis			**.002**		**.004**
Lung	7	4 (57.1)		4 (57.1)	
Other sites of metastasis	48	5 (10.4)		6 (12.5)	

Bold values indicate significant *P* values.

### Prevalence and types of *FGFR* alterations

A total of 61 *FGFR* alterations were detected, affecting 59 (16.1%) patients. Two tumors harbored 2 *FGFR* alterations each: 1 with 2 nonactionable *FGFR4* alterations (p.S430L and p.A564T) and another with 2 actionable alterations (*FGFR2* p.N549K and *FGFR3* p.Y373C). All remaining cases had either 1 or no *FGFR* alteration. Actionable *FGFR* alterations (n = 50) were identified in 49 (13.4%) patients.

**Figure 1:**
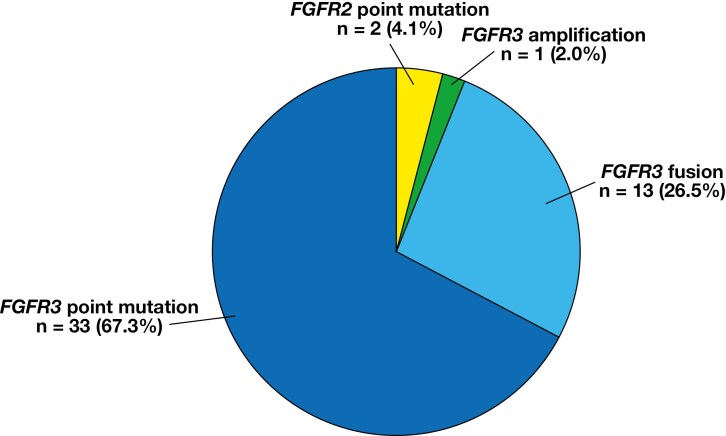
Distribution of actionable *FGFR* alterations (tiers 1 and 2).

**Figure 2:**
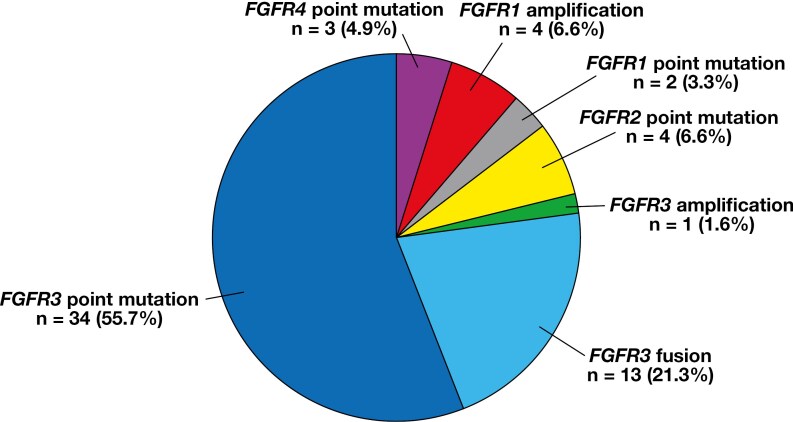
Distribution of all *FGFR* alterations (tiers 1, 2, and 3).

The distribution of *FGFR* alterations is illustrated in Figure 1 (tiers 1 and 2) and Figure 2 (tiers 1, 2, and 3). The most common types of *FGFR* alterations were *FGFR3* point mutations (n = 34) and *FGFR3::TACC3* fusions (n = 13). Other *FGFR* alterations were *FGFR1* amplification (n = 4), *FGFR1* point mutation (n = 2), *FGFR2* point mutations (n = 4), *FGFR3* amplification (n = 1), and *FGFR4* point mutations (n = 3). *FGFR* variants are detailed in Table 4.

**Table 4:** Summary of *FGFR* Variants

Variant classification	Variant	Count
Tier ½	*FGFR2* p.C382R	1
	*FGFR2* p.K659N	1
	*FGFR2* p.N549K	1
	*FGFR3* p.G375D	1
	*FGFR3* p.R248C	2
	*FGFR3* p.G370C	3
	*FGFR3* p.Y373C	7
	*FGFR3* p.S249C	20
Tier 3	*FGFR1* p.D824N	1
	*FGFR1* p.P842L	1
	*FGFR2* p.E525K	1
	*FGFR3* p.S424F	1
	*FGFR4* p.A564T	1
	*FGFR4* p.G388R	1
	*FGFR4* p.S430L	1

The most common types of *FGFR* alterations were *FGFR3* point mutations (n = 34) and *FGFR3::TACC3* fusions (n = 13). Other *FGFR* alterations were *FGFR1* amplification (n = 4), *FGFR1* point mutation (n = 2), *FGFR2* point mutations (n = 4), *FGFR3* amplification (n = 1), and *FGFR4* point mutations (n = 3). *FGFR* variants are detailed in Table 4.

Actionable FGFR alterations (n = 49) represented 82.0% of all *FGFR* alterations. There were 2.5 times more actionable *FGFR3* point mutations (n = 33) than *FGFR3* fusions (n = 13). The most common *FGFR3* variants were p.S249C (n = 20), p.Y373C (n = 7), and p.G370C (n = 3). All *FGFR3* fusions were *FGFR3::TACC3*. Three actionable *FGFR2* point mutations were detected, while no *FGFR2* fusions were identified.

### Frequency of *PIK3CA*, *TP53*, and *RB1* mutations by *FGFR* status

Analysis of a subset of *FGFR*-altered specimens (n = 33) for oncogenic (tier 1 or 2) variants in other relevant genes showed 11 (33.3%) harbored the *PIK3CA* mutation, 7 (21.2%) had the *TP53* mutation, and 1 (3.0%) had the *RB1* mutation (with concurrent *TP53* mutation). Among these, 2 demonstrated comutations in both *PIK3CA* and *TP53*.

Upon analysis of a subset of *FGFR*-negative specimens (n = 179), 27 (15.0%) were found to have the *PIK3CA* mutation, 101 (54%) had the *TP53* mutation, and 35 (19.6%) had the *RB1* mutation. *TP53* mutations co-occurred with *PIK3CA* mutations in 17 (63.0%) of 27 *PIK3CA*-mutated carcinomas. Of the 35 *RB1*-mutated specimens, 31 (88.6%) had concurrent *TP53* mutations.

The difference in the frequency of *PIK3CA*, *TP53*, and *RB* mutations between the 2 groups was significant, with a *P* value of .012, less than .001, and .020, respectively Table 5. Further details about the comutation profiles of these 212 consecutive urinary tract carcinomas are provided in the Supplementary Material.

**Table 5:** Prevalence of Oncogenic *PIK3CA*, *TP53*, and *RB1* Mutations in Urinary Tract Carcinomas With vs Without *FGFR* Alteration

Oncogenic (tier 1 or 2) *PIK3CA/TP53/RB1* variants	Specimens with any tier 1, 2, or 3 *FGFR* alteration (n = 33), No. (%)	Specimens with no tier 1, 2, or 3 *FGFR* alteration (n = 179), No. (%)	χ^2^ *P* value
*PIK3CA* mutation	11 (33.3)	27 (15.0)	.012
*TP53* mutation	7 (21.2)	101 (56.4)	<.001
*RB1* mutation	1 (3.0)	35 (19.6)	.020

### Clinicopathologic correlates of *FGFR* alterations

The most notable finding was the significantly higher prevalence of *FGFR* alterations in upper urinary tract carcinomas compared to lower urinary tract carcinomas (23.6% vs 13.4%, *P* = .037), as shown in Table 2. The difference remained significant when analyzing actionable *FGFR* alterations only (22.2% vs 10.0%, *P* = .007). All *FGFR* alterations found in upper urinary tract carcinomas were actionable *FGFR3* alterations. Compared to primary tumors, metastases had 1.3 times more actionable *FGFR* alterations, but the difference was not statistically significant (*P* = .482). Lung metastases had significantly more *FGFR* alterations than metastases of other sites (approximately 5 times more, *P* = .004). The prevalence of *FGFR* alterations was statistically comparable between pathological tumor stages. *FGFR* alterations were more frequent in specimens with negative lymph node status than in tumors with positive lymph node(s), although the difference did not reach statistical significance (*P* = .100). Regarding demographic features, actionable *FGFR* alterations exclusively occurred in patients older than 55 years (*P* = .097), while sex did not impact the positivity rate.

**Table 2:** Associations Between Clinicopathological Variables and the Prevalence of *FGFR* Alterations in Primary Urinary Tract Carcinomas (n = 311)

Characteristic	N	Actionable (tier 1 or 2) *FGFR* alteration(s), No. (%)	χ^2^ *P* value	Any (tier 1, 2, or 3) *FGFR* alteration(s), No. (%)	χ^2^ *P* value
Tumor site			**.007**		**.037**
Upper urinary tract	72	16 (22.2)		17 (23.6)	
Lower urinary tract	239	24 (10.0)		32 (13.4)	
Pathological tumor stage			.279		.221
Early stage (pTa/pT1)	42	6 (14.3)		7 (16.7)	
Muscle invasive (pT2)	101	9 (8.9)		11 (10.9)	
Locally advanced (pT3/pT4)	150	19 (12.7)		24 (15.0)	
NA	18				
Pathological nodal stage			.102		.146
Negative (pN0)	63	9 (14.3)		12 (19.0)	
Positive (pN1/pN2/pN3)	69	4 (5.8)		7 (10.1)	
NA	179				

NA, not applicable. Bold values represent significant *P* values.

### Histologic subtypes and *FGFR* alterations

While almost all sequenced specimens were urothelial carcinomas, 5 were squamous cell carcinomas, and 3 were adenocarcinomas. All *FGFR* alterations were found in urothelial carcinomas, except for 1, which occurred in a squamous cell carcinoma. However, the latter was an *FGFR1* single-nucleotide variant of uncertain clinical significance (tier 3). Actionable *FGFR* alterations were detected in pure urothelial carcinoma not otherwise specified (n = 40), urothelial carcinoma with squamous differentiation (n = 6), urothelial carcinoma with micropapillary features (n = 2), and urothelial carcinoma with nested features (n = 2). Eight of these 10 specimens with subtype features and tier 1/2 *FGFR* alteration(s) were of the lower urinary tract, and 2 were of the upper urinary tract. One urothelial carcinoma with glandular differentiation had a tier 3 *FGFR* alteration. Trophoblastic differentiation, plasmacytoid features, neuroendocrine features, sarcomatoid features, clear cell features, and microcystic features were not associated with *FGFR* alterations in this cohort.

## DISCUSSION

In this prospective, dual-institution cohort study of 366 patients with urinary tract carcinoma, somatic *FGFR* alterations were identified in 16.1% of cases, with actionable variants comprising 13.4% of the total cohort. *FGFR3* variants found in the present study are consistent with previous reports, including the 4 variants detected in the BLC2001 study (p.S249C, p.Y373C, p.R248C, and p.G370C),^8,9^ in addition to p.G375D (n = 1). In studies that assessed the frequency of *FGFR3* mutations in cohorts composed exclusively^18^ or predominantly^19^ of non-muscle-invasive bladder tumors, additional variants were found in exon 10 (p.S371C, p.A391E, p.A369E, p.I376C, p.L377L, p.G380R) and exon 15 (p.K650E, p.K650T, p.K650M), while p.G370C and p.G375D were not identified. With p.S249C, p.Y373C, and p.R248C being found in all stages, it is hypothesized that metastatic/advanced bladder cancer with these mutations results from the progression of lower-stage tumors that are already harboring these mutations. Whether these variants portend a higher risk of disease progression remains unclear. Finally, p.G370C and p.G375D variants appear to be present only in metastatic/advanced cancer, suggesting they may represent progression-related mutations, but these variants were rare, which makes their significance difficult to interpret statistically. Regarding *FGFR3* fusions, current findings showed they were significantly less frequent than *FGFR3* mutations, and *TACC3* was the fusion partner, which corroborates previously published data.^20^ Among all *FGFR* alterations reported using CAP/ASCO/AMP guidelines,^17^  *FGFR3* point mutations were the most predominant, which represents an advantage in terms of therapeutic response rate. *FGFR3* mutations have indeed been reported to be associated with a higher response rate to erdafitinib compared to *FGFR2/3* fusions (49% vs 16%) in the BCL2001 trial.^9^

Prior studies found *FGFR3* mutations to be present in approximately 70% to 80% of low-grade, low-stage papillary urothelial carcinomas, and they often occur in combination with *PIK3CA* mutations.^19,21,22^ Additionally, *CDKN2A* homozygous deletion has been shown to be linked to progression in *FGFR3*-mutated urothelial carcinoma.^23,24^ On the other hand, *TP53* and *RB* mutations are known to be driving oncogenesis in high-grade, high-stage urothelial carcinomas. With *FGFR3* and *TP53* mutations being mostly exclusive in past studies, it is believed that they correspond to 2 different pathogenetic pathways.^19,25^ The recent Consensus Molecular Classification of Muscle-Invasive Bladder Cancer by Kamoun et al,^26^ which defined 6 molecular classes based on 1750 muscle-invasive transcriptomic profiles from 17 cohorts, including 15 from published studies, showed enrichment in *TP53* mutations in the luminal unstable subtype (76%) and the basal/squamous subtype (61%), enrichment in *RB1* mutations in the luminal unstable subtype (25%) and the neuroendocrine subtype (39%), and enrichment in *FGFR3* mutations in the luminal papillary subtype (33%). Compared to the luminal unstable and basal/squamous subtypes, luminal papillary tumors (n = 120) had a lower prevalence of *TP53* mutations (32%) and *RB1* mutations (5%). In concordance with the literature, the present study identified, using next-generation sequencing, significantly more *PIK3CA* mutations and fewer *TP53* and *RB1* mutations in *FGFR*-altered vs *FGFR*-negative urothelial carcinomas. The clinical significance of these comutations in *FGFR*-altered tumors remains to be elucidated.

The present study, demonstrating that 13.4% of patients had actionable *FGFR* alterations, highlights the therapeutic potential of erdafitinib in metastatic or locally advanced urinary tract carcinomas. This proportion is concordant with the existing literature, which reports percentages between 12% and 16.6% in muscle-invasive tumors.^10,19,27^ Stratification by pathological tumor stage, however, showed comparable positivity rates between locally advanced tumors and lower-stage tumors, contrasting with results from other groups. A retrospective study from van Rhijn et al^19^ detected *FGFR3* mutations in 77% of pTa, 31% pT1, and 15% of pT2+ urothelial carcinomas, based on 260 tumors. However, these were all localized, nonmetastatic tumors and consisted of a mix of low-grade and high-grade tumors, while the lower-stage tumors analyzed in the present study represented primary samples from metastatic cases and were all of high grade. This may explain the discordance in the prevalence of *FGFR3* mutations in lower-stage (pTa/pT1) tumors between the present study and prior studies. The Cancer Genome Atlas Research Network found a *FGFR3* mutation rate of 12% upon sequencing of a retrospective cohort of 130 chemotherapy-naive, T2 to T4a high-grade urothelial carcinomas.^27^ Using a prospective cohort of metastatic or unresectable, locally advanced urothelial carcinoma, Loriot and colleagues^10^ identified actionable *FGFR2/3* alterations in 16.6% of 7293 patients. To our knowledge, the current work is the first external, prospective study evaluating the prevalence of both *FGFR2* and *FGFR3* alterations in metastatic/advanced urinary tract carcinomas since the publication of the BCL2001 study. Based on a real-world cohort of patients with a diverse ethnic background, this study is also the first, to our knowledge, to assess the benefit of integrating reflex somatic testing in the clinical workflow and the first to report the prevalence of actionable and nonactionable *FGFR1-4* alterations fulfilling CAP/ASCO/AMP reporting criteria.^17^ A prior study by Fischbach and colleagues^28^ focused on *FGFR* amplifications and found a rate of 3.4% in a cohort of 153 bladder tumors. In the present study, 10 nonactionable *FGFR1-4* variants were found, affecting 2.7% of patients. While *FGFR* alterations other than *FGFR2/3* point mutations or fusions have not yet been approved as predictive biomarkers in urinary tract carcinomas, these were reported in the anticipation of potential future evidence supporting their therapeutic value. *FGFR* alterations currently classified as nonactionable may become actionable with the use of other *FGFR* inhibitors or upon analysis of larger, multiethnic, multigeographical cohorts. Recently, the FORT-1 phase II/III trial, led by Sternberg and colleagues,^29^ demonstrated that rogaratinib and chemotherapy had comparable efficacy and safety in patients with metastatic/advanced urothelial carcinoma exhibiting *FGFR1/3* messenger RNA overexpression. The phase II RAGNAR trial demonstrated the benefit of erdafitinib in numerous non–urothelial carcinoma solid tumors harboring any activating *FGFR1-4* alterations.^30^ Besides erdafitinib, other *FGFR* inhibitors, including futibatinib, infigratinib, and pemigatinib, have been approved by the FDA for the treatment of cholangiocarcinoma^31-33^; pemigatinib has been approved for myeloid/lymphoid neoplasm.^34^ Options for *FGFR*-targeted therapy are numerous yet underexplored in urinary tract carcinomas.

The higher prevalence of *FGFR* alterations, including actionable *FGFR2/3* alterations, in upper tract carcinomas compared to lower tract carcinomas shown in the current study is in concordance with findings from Sfakianos and colleagues.^35^ This group identified *FGFR3* alterations in 35.6% of 83 upper tract carcinomas treated with nephroureterectomy and in 21.6% of 102 bladder carcinomas treated with radical cystectomy (*P* = .065). These findings underscore the particularly promising therapeutic role of erdafitinib in upper tract carcinomas and highlight the importance of including urothelial carcinomas of the upper tract when establishing eligibility criteria for pathologist-driven reflex FGFR testing.

While it has been described that the luminal papillary subtype is enriched with *FGFR3* mutations, *FGFR3* copy number gain, and *FGFR3* overexpression,^26,27,36^ no previous studies have searched for associations between *FGFR* alterations and variant histologies in metastatic/advanced urothelial carcinomas. The present study, which included urothelial carcinomas with divergent differentiation and/or subtype features, revealed actionable *FGFR* alterations in pure urothelial carcinoma, urothelial carcinoma with squamous differentiation, urothelial carcinoma with micropapillary features, and urothelial carcinoma with nested features. A few squamous cell carcinomas and adenocarcinomas were also sequenced in this study, but no actionable *FGFR* alterations were detected. Another group (Eisner et al^37^) has explored the benefit of *FGFR*-targeted therapy in high-risk non-muscle-invasive bladder (defined as the presence of carcinoma in situ, lymphovascular invasion, or variant histology) and found *FGFR* alterations in 31% of tumors. There were, however, no comparisons between the different variant histologies. Further, larger cohorts will better characterize the frequency of *FGFR* alterations in variant histologies, particularly in the aggressive subtypes, which may gain great benefit from *FGFR*-targeted therapy.

A few groups have aimed to compare the *FGFR3* status in primary urothelial carcinomas vs metastases. Guancial and colleagues^38^ found the *FGFR3* mutation in 2% of 121 primary specimens compared to 9% among 33 metastases. Major limitations were the retrospective design and the use of hotspot sequencing via OncoMap and molecular inversion probe array, which may explain the limited detection rate. Turo et al^39^ used immunohistochemistry to compare *FGFR3* expression in matched primary bladder tumors and lymph node metastases in 150 patients. *FGFR3* expression was noted in 53 primary tumors and 56 metastases. However, no molecular confirmation was conducted, and *FGFR3* immunohistochemistry has not yet been validated or approved as a predictive biomarker for erdafitinib. The present study outperforms previous ones by its prospective nature, larger cohort size, and the use of next-generation sequencing, which enabled comprehensive and robust analysis of *FGFR* alterations. It is worth noting that all studies showed marginally more *FGFR* alterations in metastases compared to primary tumors, but none of them have found any significant difference.^38,39^ Thus, there may be no benefit in repeating *FGFR* testing on metastases if the primary tumor has already been tested.

Other clinicopathologic variables analyzed in this study included age, metastatic sites, and pathological nodal stage. Results suggest there may be no relevance in conducting somatic *FGFR* testing in patients younger than 55 years as we found no *FGFR* alterations in this age group. On another note, the greater rate of *FGFR* alterations in lung metastases compared to metastases of other sites suggests that *FGFR* alterations can affect the pattern of metastatic spread. An understanding of this metastatic pattern may better guide clinical investigation and management. Finally, the regional lymph node status does not appear to be correlated with the frequency of *FGFR* alteration in primary tumors. These findings remain to be validated in further cohorts.

The main limitations of this study were the lack of paired primary and metastatic specimens from the same patients and the relatively small cohort size, despite this being the largest study to date on reflex *FGFR* somatic testing—a practice that remains relatively new in clinical settings. Since this study reports an experience based on prospective real-world cohorts under a publicly funded health system, only 1 sample was sequenced per patient for cost-effectiveness. Obtaining the molecular profile of all primary and metastatic samples from a same patient may be more informative. Additionally, the origin of metastases (lower vs upper urinary tract) was not specified in this study since the clinical history was not always available, especially for external specimens referred from outside institutions. Separating carcinomas of upper tract origin from carcinomas of lower tract origin when comparing metastases to primary tumors would provide more accurate results. The composite percentage of histologic variants in the circled tumor sent for molecular analysis was also not recorded. A low percentage can indeed lead to false-negative results and underestimate the positivity rate associated with variant histologies. Microdissection to separate tumor components would increase the accuracy of histologic correlates but may lead to insufficient DNA due to the relatively low yield of such a technique. Finally, differences based on ethnicity could not be explored, given that these data were unavailable in the electronic patient records.

Future studies that include a larger cohort will validate the clinicopathologic correlates of *FGFR*-altered urinary tract carcinomas described herein and assess response to FGFR inhibitor therapy through long-term follow-up of clinical outcomes.

## CONCLUSIONS

The experience of 2 institutions incorporating reflex somatic *FGFR* testing in metastatic or locally advanced (pT3/4) urinary tract carcinomas was reported. Based on a prospective real-world cohort of 366 urinary tract carcinomas, the detection rate for *FGFR* alterations was 16.1%. Actionable *FGFR* alterations were detected in 13.4% of patients. Upper tract urothelial carcinomas and lung metastases were enriched in *FGFR* alterations, compared to lower tract carcinomas and metastases of other sites. Metastases had marginally more *FGFR* alterations compared to primary specimens. *FGFR* alterations were seen in pure urothelial carcinomas and certain variant histologies. Younger patients (<55 years) did not harbor *FGFR* alterations in our cohorts. Further cohorts are required to validate the clinicopathologic associations observed herein. The findings showcased in this study may help establish guidelines for predictive somatic *FGFR* testing in urinary tract carcinomas and shape therapeutic strategies.

## Supplementary Material

aqaf108_Supplementary_Material

## References

[1] Siegel RL, Giaquinto AN, Jemal A. Cancer statistics, 2024 [published correction appears in CA Cancer J Clin. 2024;74(2):203]. CA Cancer J Clin. 2024;74:12-49. doi: 10.3322/caac.2182038363123

[2] EAU Guidelines. Edn. presented at the EAU Annual Congress Madrid 2025. EAU Guidelines Office, Arnhem, The Netherlands. ISBN 978-94-92671-29-5. Accessed June 5, 2025. http://uroweb.org/guidelines/compilations-of-all-guidelines/

[3] Yafi FA, Aprikian AG, Chin JL, et al Contemporary outcomes of 2287 patients with bladder cancer who were treated with radical cystectomy: a Canadian multicentre experience. BJU Int. 2011;108:539-545. doi: https://doi.org/10.1111/j.1464-410X.2010.09912.x 21166753

[4] Stecca C, Abdeljalil O, Sridhar SS. Metastatic urothelial cancer: a rapidly changing treatment landscape. Ther Adv Med Oncol. 2021;13:17588359211047352. doi: https://doi.org/10.1177/17588359211047352 34616491 PMC8488509

[5] National Comprehensive Cancer Network. Bladder Cancer: NCCN Guidelines Version 1.2025. Updated March 25, 2025. Accessed June 5, 2025. https://www.nccn.org/professionals/physician_gls/pdf/bladder.pdf

[6] Haugsten EM, Wiedlocha A, Olsnes S, Wesche J. Roles of fibroblast growth factor receptors in carcinogenesis. Mol Cancer Res. 2010;8:1439-1452. doi: https://doi.org/10.1158/1541-7786.MCR-10-0168 21047773

[7] Roskoski R Jr . The role of fibroblast growth factor receptor (FGFR) protein-tyrosine kinase inhibitors in the treatment of cancers including those of the urinary bladder. Pharmacol Res. 2020;151:104567. doi: https://doi.org/10.1016/j.phrs.2019.104567 31770593

[8] Katoh M, Loriot Y, Brandi G, Tavolari S, Wainberg ZA, Katoh M. FGFR-targeted therapeutics: clinical activity, mechanisms of resistance and new directions. Nat Rev Clin Oncol. 2024;21:312-329. doi: https://doi.org/10.1038/s41571-024-00869-z 38424198

[9] Loriot Y, Necchi A, Park SH, et al; BLC2001 Study Group. Erdafitinib in locally advanced or metastatic urothelial carcinoma. N Engl J Med. 2019;381:338-348. doi: https://doi.org/10.1056/NEJMoa1817323 31340094

[10] Loriot Y, Matsubara N, Park SH, et al; THOR Cohort 1 Investigators. Erdafitinib or chemotherapy in advanced or metastatic urothelial carcinoma. N Engl J Med. 2023;389:1961-1971. doi: https://doi.org/10.1056/NEJMoa2308849 37870920

[11] Siefker-Radtke AO, Necchi A, Park SH, et al; BLC2001 Study Group. Efficacy and safety of erdafitinib in patients with locally advanced or metastatic urothelial carcinoma: long-term follow-up of a phase 2 study. Lancet Oncol. 2022;23:248-258. doi: https://doi.org/10.1016/S1470-2045(21)00660-4 35030333

[12] Food and Drug Administration. FDA grants accelerated approval to erdafitinib for metastatic urothelial carcinoma. Updated April 12, 2025. Accessed June 5, 2025. https://www.fda.gov/drugs/resources-information-approved-drugs/fda-grants-accelerated-approval-erdafitinib-metastatic-urothelial-carcinoma

[13] Government of Canada. Summary basis of decision for Balversa. Updated January 28, 2020. Accessed June 5, 2025. https://dhpp.hpfb-dgpsa.ca/review-documents/resource/SBD00459

[14] Cancer Care Ontario. Comprehensive Cancer Biomarker Testing Program. Updated April 1, 2025. Accessed June 5, 2025. https://www.cancercareontario.ca/en/guidelines-advice/treatment-modality/pathology-laboratory-testing/genetic-testing-resources/comprehensive-cancer-biomarker-testing-program

[15] Food and Drug Administration. FDA approves erdafitinib for locally advanced or metastatic urothelial carcinoma. Updated January 19, 2024. Accessed June 5, 2025. https://www.fda.gov/drugs/resources-information-approved-drugs/fda-approves-erdafitinib-locally-advanced-or-metastatic-urothelial-carcinoma

[16] Canada’s Drug Agency. Erdafitinib. Updated May 27, 2025. Accessed June 5, 2025. https://www.cda-amc.ca/erdafitinib

[17] Li MM, Datto M, Duncavage EJ, et al Standards and guidelines for the interpretation and reporting of sequence variants in cancer: a joint consensus recommendation of the Association for Molecular Pathology, American Society of Clinical Oncology, and College of American Pathologists. J Mol Diagn. 2017;19:4-23. doi: https://doi.org/10.1016/j.jmoldx.2016.10.002 27993330 PMC5707196

[18] Hernández S, López-Knowles E, Lloreta J, et al Prospective study of FGFR3 mutations as a prognostic factor in nonmuscle invasive urothelial bladder carcinomas. J Clin Oncol. 2006;24:3664-3671. doi: https://doi.org/10.1200/JCO.2005.05.1771 16877735

[19] van Rhijn BW, van der Kwast TH, Vis AN, et al FGFR3 and P53 characterize alternative genetic pathways in the pathogenesis of urothelial cell carcinoma. Cancer Res. 2004;64:1911-1914. doi: https://doi.org/10.1158/0008-5472.can-03-2421 15026322

[20] Robertson AG, Kim J, Al-Ahmadie H, et al; TCGA Research Network. Comprehensive molecular characterization of muscle-invasive bladder cancer [published correction appears in Cell. 2018;174(4):1033]. Cell. 2017;171:540-556.e25. doi: 10.1016/j.cell.2017.09.007 28988769 10.1016/j.cell.2017.09.007 PMC5687509

[21] Billerey C, Chopin D, Aubriot-Lorton MH, et al Frequent FGFR3 mutations in papillary non-invasive bladder (pTa) tumors. Am J Pathol. 2001;158:1955-1959. doi: https://doi.org/10.1016/S0002-9440(10)64665-2 11395371 PMC1891972

[22] López-Knowles E, Hernández S, Malats N, et al PIK3CA mutations are an early genetic alteration associated with FGFR3 mutations in superficial papillary bladder tumors. Cancer Res. 2006;66:7401-7404. doi: https://doi.org/10.1158/0008-5472.CAN-06-1182 16885334

[23] Rebouissou S, Hérault A, Letouzé E, et al CDKN2A homozygous deletion is associated with muscle invasion in FGFR3-mutated urothelial bladder carcinoma. J Pathol. 2012;227:315-324. doi: https://doi.org/10.1002/path.4017 22422578

[24] Downes MR, Weening B, van Rhijn BW, Have CL, Treurniet KM, van der Kwast TH. Analysis of papillary urothelial carcinomas of the bladder with grade heterogeneity: supportive evidence for an early role of CDKN2A deletions in the FGFR3 pathway. Histopathology. 2017;70:281-289. doi: https://doi.org/10.1111/his.13063 27530957

[25] Bakkar AA, Wallerand H, Radvanyi F, et al FGFR3 and TP53 gene mutations define two distinct pathways in urothelial cell carcinoma of the bladder. Cancer Res. 2003;63:8108-8112. 14678961

[26] Kamoun A, de Reyniès A, Allory Y, et al; Bladder Cancer Molecular Taxonomy Group. A consensus molecular classification of muscle-invasive bladder cancer. Eur Urol. 2020;77:420-433. doi: https://doi.org/10.1016/j.eururo.2019.09.006 31563503 PMC7690647

[27] Cancer Genome Atlas Research Network. Comprehensive molecular characterization of urothelial bladder carcinoma. Nature. 2014;507:315-322. doi: https://doi.org/10.1038/nature12965 24476821 PMC3962515

[28] Fischbach A, Rogler A, Erber R, et al Fibroblast growth factor receptor (FGFR) gene amplifications are rare events in bladder cancer. Histopathology. 2015;66:639-649. doi: https://doi.org/10.1111/his.12473 24898159

[29] Sternberg CN, Petrylak DP, Bellmunt J, et al FORT-1: phase II/III study of rogaratinib versus chemotherapy in patients with locally advanced or metastatic urothelial carcinoma selected based on *FGFR1*/*3* mRNA expression. J Clin Oncol. 2023;41:629-639. doi: https://doi.org/10.1200/JCO.21.02303 36240478 PMC9870218

[30] Pant S, Schuler M, Iyer G, et al; RAGNAR Investigators. Erdafitinib in patients with advanced solid tumours with FGFR alterations (RAGNAR): an international, single-arm, phase 2 study. Lancet Oncol. 2023;24:925-935. doi: https://doi.org/10.1016/S1470-2045(23)00275-9 37541273 PMC11224843

[31] Goyal L, Meric-Bernstam F, Hollebecque A, et al; FOENIX-CCA2 Study Investigators. Futibatinib for *FGFR2*-rearranged intrahepatic cholangiocarcinoma. N Engl J Med. 2023;388:228-239. doi: https://doi.org/10.1056/NEJMoa2206834 36652354

[32] Javle M, Roychowdhury S, Kelley RK, et al Infigratinib (BGJ398) in previously treated patients with advanced or metastatic cholangiocarcinoma with FGFR2 fusions or rearrangements: mature results from a multicentre, open-label, single-arm, phase 2 study. Lancet Gastroenterol Hepatol. 2021;6:803-815. doi: https://doi.org/10.1016/S2468-1253(21)00196-5 34358484

[33] Abou-Alfa GK, Sahai V, Hollebecque A, et al Pemigatinib for previously treated, locally advanced or metastatic cholangiocarcinoma: a multicentre, open-label, phase 2 study [published correction appears in Lancet Oncol. 2024;25(1):e3]. Lancet Oncol. 2020;21:671-684. doi: https://doi.org/10.1016/S1470-2045(20)30109-1 PMC8461541 32203698

[34] Gotlib J, Kiladjian J-J, Vannucchi A, et al A phase 2 study of pemigatinib (FIGHT-203; INCB054828) in patients with myeloid/lymphoid neoplasms (MLNs) with fibroblast growth factor receptor 1 (FGFR1) rearrangement. Blood. 2021;138:385-385. doi: https://doi.org/10.1182/blood-2021-148103

[35] Sfakianos JP, Cha EK, Iyer G, et al Genomic characterization of upper tract urothelial carcinoma. Eur Urol. 2015;68:970-977. doi: https://doi.org/10.1016/j.eururo.2015.07.039 26278805 PMC4675454

[36] Marzouka NA, Eriksson P, Rovira C, Liedberg F, Sjödahl G, Höglund M. A validation and extended description of the Lund taxonomy for urothelial carcinoma using the TCGA cohort. Sci Rep. 2018;8:3737. doi: https://doi.org/10.1038/s41598-018-22126-x 29487377 PMC5829240

[37] Eisner JR, de Jong FC, Shibata Y, et al Characterization of FGFR alterations and activation in patients with high-risk non-muscle-invasive bladder cancer. Clin Cancer Res. 2024;30:5374-5384. doi: https://doi.org/10.1158/1078-0432.CCR-24-2015 39325014

[38] Guancial EA, Werner L, Bellmunt J, et al FGFR3 expression in primary and metastatic urothelial carcinoma of the bladder. Cancer Med. 2014;3:835-844. doi: https://doi.org/10.1002/cam4.262 24846059 PMC4303151

[39] Turo R, Harnden P, Thygesen H, et al FGFR3 expression in primary invasive bladder cancers and matched lymph node metastases. J Urol. 2015;193:325-330. doi: https://doi.org/10.1016/j.juro.2014.06.026 24933362

