# Supplementary material for: Reflex somatic testing for the detection of FGFR alterations in urinary tract carcinomas: A dual-institutional experience
Source: Am J Clin Pathol. 2025 Oct 16;164(6):861–9. doi: 10.1093/ajcp/aqaf108 (PMC12782305; doi:10.1093/ajcp/aqaf108)
Supplement: aqaf108_Supplementary_Material [file aqaf108_supplementary_material.docx]

**Supplementary Table S1.** Summary of *PIK3CA* variants in 212 consecutive urinary tract carcinomas

| ***PIK3CA* variant** | **Count** |
| --- | --- |
| p.E545K | 17 (B) |
| p.E542K | 9 |
| p.E726K | 3 (B) |
| p.G118D | 2 (B) |
| p.M1043I | 2 |
| p.Q546K | 2 (F) |
| p.D549H | 1 (F) |
| p.E543K | 1 |
| p.E545A | 1 (F) |
| p.H1047R | 1 |
| p.P539R | 1 (F) |
| p.Q546E | 1 |
| p.R88Q | 1 |
| p.V344M | 1 |
| TOTAL | 43 |

The MANE Select transcript (NM_006218.4) was used for *PIK3CA* variant annotations. (B)=seen in both *FGFR*-altered and *FGFR*-negative specimens; (F)=seen in *FGFR*-altered specimen(s) only.

**Supplementary table S2.** Summary of *TP53* variants in 212 consecutive urinary tract carcinomas

| ***TP53* variant** | **Count** | ***TP53* variant** | **Count** |
| --- | --- | --- | --- |
| p.E285K | 15 | p.M243Afs*14 | 1 |
| p.R175H | 6 (B) | p.P152L | 1 |
| p.R280T | 5 | p.P190L | 1 |
| p.R248Q | 4 | p.P278H | 1 |
| p.E271K | 3 | p.P278L | 1 |
| p.E286K | 3 | p.P278R | 1 |
| p.Q192* | 3 | p.Q100* | 1 |
| p.R213* | 3 (B) | p.Q192Sfs*17 | 1 |
| p.R280K | 3 | p.R209Kfs*6 | 1 |
| c.673-9_675delATCTCCTAGGTT (p.?) | 2 | p.R213L | 1 |
| p.C135F | 2 | p.R213Q | 1 |
| p.C176F | 2 | p.R248W | 1 (F) |
| p.C275F | 2 | p.R273H | 1 |
| p.Q331* | 2 | p.R273L | 1 |
| p.R248L | 2 | p.R273S | 1 |
| p.R273C | 2 | p.S127F | 1 |
| p.S241Y | 2 | p.S127Y | 1 |
| p.Y220C | 2 | p.S215I | 1 |
| p.[G293=;E294*] | 1 | p.S241C | 1 |
| p.C229* | 1 (F) | p.S241F | 1 |
| p.C238G | 1 | p.S90Gfs*32 | 1 |
| p.C242Y | 1 | p.T118Qfs*5 | 1 |
| p.C527F | 1 (F) | p.T125R | 1 |
| p.D228H | 1 | p.V274_C277del | 1 |
| p.D281H | 1 | p.W91Cfs*32 | 1 |
| p.E171* | 1 | p.Y220_P223del | 1 |
| p.E258K | 1 | (NM_000546.5) p.[L257=;E258K] | 1 |
| p.E346* | 1 | (NM_000546.5) p.E198Kfs*49 | 1 |
| p.E349* | 1 | (NM_000546.5) p.I255del | 1 |
| p.F113V | 1 (F) | (NM_000546.5) p.S106Rfs*43 | 1 |
| p.F134L | 1 | c.373_375+6delACGGTCAGT (p.?) | 1 |
| p.G199E | 1 | c.783-2A>C (p.?) | 1 |
| p.G245D | 1 | c.783-2A>G (p.?) | 1 |
| p.G245S | 1 | c.919+1G>T (p.?) | 1 |
| p.G279V | 1 | c.994-1G>C (p.?) | 1 |
| p.H178Tfs*69 | 1 | p.E258G | 1 |
| p.H179Y | 1 | p.E285* | 1 (F) |
| p.I195T | 1 | p.K120E | 1 |
| p.K351* | 1 | p.K132* | 1 |
| p.L265R | 1 | TOTAL | 125 |
| p.M237I | 1 |  |  |

The MANE Select transcript (NM_000546.6) was used for TP53 variant annotations, unless otherwise specified. (B)=seen in both *FGFR*-altered and *FGFR*-negative specimens; (F)=seen in a *FGFR*-altered specimen.

**Supplementary Table S3.** Summary of *RB1* variants in 212 consecutive urinary tract carcinomas

| ***RB1* variant** | **Count** |
| --- | --- |
| c.2490-1G>A (p.?) | 2 |
| p.A18Pfs*3 | 1 |
| p.D286* | 1 |
| p.D346Efs*3 | 1 |
| p.E50* | 1 |
| p.G437Vfs*20 | 1 |
| p.I101Sfs*10 | 1 |
| p.I831Nfs*7 | 1 |
| p.K870* | 1 |
| p.K95* | 1 |
| p.N399Kfs*7 | 1 (F) |
| p.P793* | 1 |
| p.Q471* | 1 |
| p.Q504* | 1 |
| p.Q685* | 1 |
| p.Q702* | 1 |
| p.Q736* | 1 |
| p.R251* | 1 |
| p.R445* | 1 |
| p.R556* | 1 |
| p.R579* | 1 |
| p.R787* | 1 |
| p.S230* | 1 |
| p.S249* | 1 |
| p.S463* | 1 |
| p.S576* | 1 |
| p.S829* | 1 |
| p.T377Sfs*17 | 1 |
| p.T664Nfs*4 | 1 |
| p.V190Gfs*2 | 1 |
| p.Y321* | 1 |
| (NM_000321.2) p.E843* | 1 |
| (NM_000321.2) p.L335* | 1 |
| c.2104_2106+10delCAAGTAAGAAAAT (p.?) | 1 |
| c.2107-1G>A (p.?) | 1 |
| c.2520+3G>C (p.?) | 1 |
| TOTAL | 37 |

The MANE Select transcript (NM_000321.3) was used for *RB1* variant annotations, unless otherwise specified. (F)=seen in a *FGFR*-altered specimen.

**Supplementary table S4**. Summary of co-mutation profiles of 212 consecutive urinary tract carcinomas with or without *FGFR* alteration

|  | **Specimens with any Tier 1, 2 or 3 *FGFR* alteration (N=33)** | **Specimens with no Tier 1, 2 or 3  *FGFR* alteration (N=179)** |
| --- | --- | --- |
| *PIK3CA-, TP53-, RB1-* | 17 (51.5%) | 63 (35.2%) |
| *PIK3CA+, TP53-, RB1-* | 9 (27.3%) | 11 (6.1%) |
| *PIK3CA+, TP53+, RB1-* | 2 (6.1%) | 11 (6.1%) |
| *PIK3CA+, TP53+, RB1+* | 0 (0%) | 5 (2.3%) |
| *PIK3CA-, TP53+, RB1-* | 4 (12.1%) | 59 (33.0%) |
| *PIK3CA-, TP53+, RB1+* | 1 (3.0%) | 26 (14.5%) |
| *PIK3CA-, TP53-, RB1+* | 0 (0%) | 4 (2.2%) |

**Supplementary Table S5**. Detailed molecular profiles of 212 consecutive urinary tract carcinomas

| **Study specimen ID** | **Tier 1, 2 or 3 *FGFR* alteration** | **Tier 1 or 2 *PIK3CA / TP53 / RB1* mutation(s)** |
| --- | --- | --- |
| 1 | *FGFR3* p.S249C | *PIK3CA* p.E545K, *PIK3CA* p.D549H |
| 2 | *FGFR3* p.S249C | *PIK3CA* p.E545K, *PIK3CA* p.E726K |
| 3 | *FGFR3* p.Y373C | *PIK3CA* p.G118D, *PIK3CA* p.K111N |
| 4 | *FGFR3* p.G370C | *PIK3CA* p.E545K |
| 5 | *FGFR3* p.S249C | *PIK3CA* p.E545K |
| 6 | *FGFR3* p.S249C | *PIK3CA* p.E545K |
| 7 | *FGFR3::TACC3* | *PIK3CA* p.E545K |
| 8 | *FGFR3* p.S249C | *PIK3CA* p.P539R |
| 9 | *FGFR3* p.S249C | *PIK3CA* p.Q546K |
| 10 | *FGFR2* p.E525K | *PIK3CA* p.Q546K, *TP53* p.C229* |
| 11 | *FGFR3* p.S249C | *PIK3CA* p.E545A, *TP53* p.R175H |
| 12 | *FGFR1* amplification | *TP53* p.E285K, *RB1* p.N399Kfs*7 |
| 13 | *FGFR3* p.S249C | *TP53* p.R248W |
| 14 | *FGFR3* p.S249C | *TP53* p.R213* |
| 15 | *FGFR3* p.S249C | *TP53* p.F113V |
| 16 | *FGFR3::TACC3* | *TP53* p.C527F |
| 17 | *FGFR1* amplification | Not detected |
| 18 | *FGFR2* p.C382R | Not detected |
| 19 | *FGFR3* p.G370C | Not detected |
| 20 | *FGFR3* p.R248C | Not detected |
| 21 | *FGFR3* p.S249C | Not detected |
| 22 | *FGFR3* p.S249C | Not detected |
| 23 | *FGFR3* p.S249C | Not detected |
| 24 | *FGFR3* p.S249C | Not detected |
| 25 | *FGFR3* p.S249C | Not detected |
| 26 | *FGFR3* p.S249C | Not detected |
| 27 | *FGFR3* p.S249C | Not detected |
| 28 | *FGFR3* p.S249C | Not detected |
| 29 | *FGFR3* p.Y373C | Not detected |
| 30 | *FGFR3::TACC3* | Not detected |
| 31 | *FGFR3::TACC3* | Not detected |
| 32 | *FGFR3::TACC3* | Not detected |
| 33 | *FGFR3::TACC3* | Not detected |
| 34 | Not detected | *PIK3CA* p.E542K, *PIK3CA* E545K |
| 35 | Not detected | *PIK3CA* p.E542K |
| 36 | Not detected | *PIK3CA* p.E542K |
| 37 | Not detected | *PIK3CA* p.E542K |
| 38 | Not detected | *PIK3CA* p.E542K |
| 39 | Not detected | *PIK3CA* p.E545K |
| 40 | Not detected | *PIK3CA* p.E545K |
| 41 | Not detected | *PIK3CA* p.E545K |
| 42 | Not detected | *PIK3CA* p.E545K |
| 43 | Not detected | *PIK3CA* p.M1043I |
| 44 | Not detected | *PIK3CA* p.G118D |
| 45 | Not detected | *PIK3CA* p.E543K, *PIK3CA* p.M1043I, *TP53* p.E285K, *TP53* p.H178Tfs*69, *RB1* p.S249* |
| 46 | Not detected | *PIK3CA* p.E545K, *TP53* p.K120E, RB1 p.S463* |
| 47 | Not detected | *PIK3CA* p.E726K, *TP53* p.E285* |
| 48 | Not detected | *PIK3CA* p.E543K, *TP53* p.E285K, *TP53* p.S215I |
| 49 | Not detected | *PIK3CA* p.E542K, *TP53* p.E285K, *RB1* p.Q504* |
| 50 | Not detected | *PIK3CA* p.H1047R, *TP53* p.R175H, *RB1* p.R787* |
| 51 | Not detected | *PIK3CA* p.E542K, *PIK3CA* p.E545K, *TP53* p.R248Q |
| 52 | Not detected | *PIK3CA* p.E542K, *TP53* p.G199E |
| 53 | Not detected | *PIK3CA* p.Q546E, *TP53* p.M243Afs*14 |
| 54 | Not detected | *PIK3CA* p.R88Q, *TP53* p.I195T |
| 55 | Not detected | *PIK3CA* p.V344M, *TP53* p.R248Q |
| 56 | Not detected | *PIK3CA* p.E542K, *TP53* p.E285K |
| 57 | Not detected | *PIK3CA* p.E545K, *TP53* p.E349* |
| 58 | Not detected | *PIK3CA* p.E545K, *TP53* p.G245S |
| 59 | Not detected | *PIK3CA* p.E545K, *TP53* p.R280T |
| 60 | Not detected | *PIK3CA* p.E726K, *TP53* p.Q100* |
| 61 | Not detected | *TP53* p.R175H, *TP53* p.R213L, *TP53* p.R280T, *RB1* p.R445* |
| 62 | Not detected | *TP53* p.D228H, *TP53* p.E285K, *RB1* p.S576* |
| 63 | Not detected | *TP53* p.H179R, *TP53* p.R280K, *RB* (NM_000321.2) p.E843* |
| 64 | Not detected | *TP53* p.R175H, *TP53* p.R213*, *RB1* p.A18Pfs*3 |
| 65 | Not detected | *TP53* p.Y220C, *TP53* p.R280K, *RB1* p.Q471* |
| 66 | Not detected | *TP53* p.L265R, *RB1* p.R579*, *RB1* p.P793* |
| 67 | Not detected | *TP53* p.D281H, *RB1* p.I831Nfs*7 |
| 68 | Not detected | TP53 p.E258G, RB1 c.2520+3G>C (p.?) |
| 69 | Not detected | *TP53* p.E285K, *RB1* p.Y321* |
| 70 | Not detected | *TP53* p.E285K, *RB1* p.Q685* |
| 71 | Not detected | *TP53* p.E285K, *RB1* p.S230* |
| 72 | Not detected | *TP53* p.E285K, *RB1* p.T664Nfs*4 |
| 73 | Not detected | *TP53* p.E286K, *RB1* c.2490-1G>A (p.?) |
| 74 | Not detected | *TP53* p.M237I, *RB1* c.2490-1G>A (p.?) |
| 75 | Not detected | *TP53* p.P278H, *RB1* p.Q736* |
| 76 | Not detected | *TP53* p.P278R, *RB1* p.R251* |
| 77 | Not detected | *TP53* p.P152L, *RB1* p.E50* |
| 78 | Not detected | *TP53* p.R248L, *RB1* (NM_000321.2) p.L335* |
| 79 | Not detected | *TP53* p.R248Q, *RB1* p.Q702* |
| 80 | Not detected | *TP53* p.R248Q, *RB1* c.2107-1G>A (p.?) |
| 81 | Not detected | *TP53* p.R280K, *RB1* p.K95* |
| 82 | Not detected | *TP53* p.S127F, *RB1* p.D346Efs*3 |
| 83 | Not detected | *TP53* p.T118Qfs*5, *RB1* p.T377Sfs*17 |
| 84 | Not detected | *TP53* p.W91Cfs*32, *RB1* p.K870* |
| 85 | Not detected | *TP53* c.783-2A>G (p.?), *RB1* c.2104_2106+10delCAAGTAAGAAAAT (p.?) |
| 86 | Not detected | *TP53* c.783-2A>C (p.?), *RB1* p.D286* |
| 87 | Not detected | *TP53* p.E171*, *TP53* p.V274_C277del |
| 88 | Not detected | *TP53* p.E258K, *TP53* p.E271K |
| 89 | Not detected | *TP53* p.E285K, *TP53* p.R175H |
| 90 | Not detected | *TP53* p.E285K, *TP53* (NM_000546.5) p.[L257=;E258K] |
| 91 | Not detected | *TP53* p.G245D, *TP53* p.C238G |
| 92 | Not detected | *TP53* p.Q331*, *TP53* p.C275F |
| 93 | Not detected | *TP53* p.R280T, *TP53* p.E271K |
| 94 | Not detected | *TP53* p.S127Y, *TP53* (NM_000546.5) p.I255del |
| 95 | Not detected | *TP53* p.S241Y, *TP53* p.R209Kfs*6 |
| 96 | Not detected | *TP53* p.Y220C, *TP53* p.G279V |
| 97 | Not detected | *TP53* p.C135F |
| 98 | Not detected | *TP53* p.C135F |
| 99 | Not detected | *TP53* p.C176F |
| 100 | Not detected | *TP53* p.C176F |
| 101 | Not detected | *TP53* p.C242Y |
| 102 | Not detected | *TP53* p.C275F |
| 103 | Not detected | *TP53* p.E271K |
| 104 | Not detected | *TP53* p.E285K |
| 105 | Not detected | *TP53* p.E285K |
| 106 | Not detected | *TP53* p.E285K |
| 107 | Not detected | *TP53* p.E286K |
| 108 | Not detected | *TP53* p.E286K |
| 109 | Not detected | *TP53* p.E346* |
| 110 | Not detected | *TP53* p.F134L |
| 111 | Not detected | *TP53* p.H179Y |
| 112 | Not detected | *TP53* p.K132* |
| 113 | Not detected | *TP53* p.K351* |
| 114 | Not detected | *TP53* p.P190L |
| 115 | Not detected | *TP53* p.P278L |
| 116 | Not detected | *TP53* p.Q192* |
| 117 | Not detected | *TP53* p.Q192* |
| 118 | Not detected | *TP53* p.Q192* |
| 119 | Not detected | *TP53* p.Q192Sfs*17 |
| 120 | Not detected | *TP53* p.Q331* |
| 121 | Not detected | *TP53* p.R175H |
| 122 | Not detected | *TP53* p.R213* |
| 123 | Not detected | *TP53* p.R213Q |
| 124 | Not detected | *TP53* p.R248L |
| 125 | Not detected | *TP53* p.R273C |
| 126 | Not detected | *TP53* p.R273C |
| 127 | Not detected | *TP53* p.R273H |
| 128 | Not detected | *TP53* p.R273L |
| 129 | Not detected | *TP53* p.R273S |
| 130 | Not detected | *TP53* p.R280T |
| 131 | Not detected | *TP53* p.R280T |
| 132 | Not detected | *TP53* p.S241C |
| 133 | Not detected | *TP53* p.S241F |
| 134 | Not detected | *TP53* p.S241Y |
| 135 | Not detected | *TP53* p.S90Gfs*32 |
| 136 | Not detected | *TP53* p.T125R |
| 137 | Not detected | *TP53* p.Y220_P223del |
| 138 | Not detected | *TP53* p.[G293=;E294*] |
| 139 | Not detected | *TP53* (NM_000546.5) p.E198Kfs*49 |
| 140 | Not detected | *TP53* (NM_000546.5) p.S106Rfs*43 |
| 141 | Not detected | *TP53* c.373_375+6delACGGTCAGT (p.?) |
| 142 | Not detected | *TP53* c.673-9_675delATCTCCTAGGTT (p.?) |
| 143 | Not detected | *TP53* c.673-9_675delATCTCCTAGGTT (p.?) |
| 144 | Not detected | *TP53* c.919+1G>T (p.?) |
| 145 | Not detected | *TP53* c.994-1G>C (p.?) |
| 146 | Not detected | *RB1* p.G437Vfs*20 |
| 147 | Not detected | *RB1* p.V190Gfs*2 |
| 148 | Not detected | *RB1* p.R556* |
| 149 | Not detected | *RB1* p.S829* |
| 150-212 | Not detected | Not detected |

Variant annotations were based on the MANE Select transcripts (NM_000142.5 for *FGFR* variants, NM_006218.4 for *PIK3CA* variants, NM_000546.6 for *TP53* variants, and NM_000321.3 for *RB1* variants), unless otherwise specified.
